# Retraction Note: The effect of probiotics supplementation on blood pressure: a systemic review and meta-analysis

**DOI:** 10.1186/s12944-024-02418-0

**Published:** 2024-12-23

**Authors:** Dan Qi, Xiao-Lu Nie, Jian-Jun Zhang

**Affiliations:** 1 Department of Cardiology, Beijing Chao-Yang Hospital, No. 8 Gongti South Road, Chaoyang District, Beijing, 100043 China; 2 Children’s Hospital, Capital Medical University, Beijing, China

**Retraction Note: Lipids in Health and Disease (2020) 19:79**

10.1186/s12944-020-01259-x

The Editors-in-Chief have retracted this article. After publication, concerns were raised regarding the statistical analysis in the article. Specifically, it is stated that the authors used the “Begger’s test”, which is not described in the literature. The Editors-in-Chief therefore no longer have confidence in the reported data.

The authors have not replied to correspondence from the Publisher.

